# Towards responsible resource utilization: A review of sustainable vs. unsustainable reuse of wood waste

**DOI:** 10.1371/journal.pone.0312527

**Published:** 2024-12-23

**Authors:** Eric Agyemang, Kwadwo Ofori-Dua, Peter Dwumah, John Boulard Forkuor

**Affiliations:** Department of Sociology and Social Work, Kwame Nkrumah University of Science and Technology, Kumasi, Ghana; Universiti Teknologi Petronas: Universiti Teknologi PETRONAS, MALAYSIA

## Abstract

Abundant wood waste is generated globally, but the literature lacks a framework distinguishing sustainable versus unsustainable reuse practices. This gap hinders policy makers and stakeholders from effectively supporting responsible resource utilization. As such, this scoping review aimed to address this gap by evaluating wood waste reuse practices through ecological, financial, and social sustainability lenses. A comprehensive database search yielded 1,150 records, narrowed to 106 included studies through eligibility screening. Data on study details and sustainability factors was extracted without a formal quality appraisal. The protocol ensures a rigorous evidence-mapping approach. The findings revealed that sustainable uses included renewable energy, adsorbents, construction materials, and composting applications. However, toxic preservatives, uncontrolled emissions from burning, intensive harvesting impacts, and contamination risks from uncontrolled mulching perpetuate ecological, social, and financial challenges. Preventing contamination and managing sustainability trade-offs are key priorities. Research innovations, stringent quality control, and supportive policies are imperative to distinguish practices aligned with sustainability principles from those inadvertently causing harm. This review provides a comprehensive framework for making informed decisions to progress wood waste systems toward responsible resource utilization.

## Introduction

In an era where human activities are increasingly depleting natural resources, the relevance of responsible resource utilization has become an important concern for sustainable development. For this reason, the United Nations’ Sustainable Development Goal 13 is focused on taking urgent action to combat climate change and its impacts [1]. The aim is to reduce greenhouse gas emissions, increase resilience to climate hazards, and promote sustainable development pathways [2]. The judicious management of resources will ensure availability for present and future generations and safeguard the delicate balance of our ecosystem [3]. Wood, in its various forms, is one such resource that holds significant importance due to its versatility and wide range of applications. As a natural resource, it is utilized in all facets of life, making it an essential resource in today’s world [4]. For instance, its flexibility and durability make it an essential choice for commercial and residential buildings, as it is used for roofing and flooring in the building and construction industry [5]. Further, wood has been widely used in furniture and paper production, including books, newspapers, and packaging materials [6, 7]. The use of wood reveals how important it is to our way of life. That being said, the global demand for wood has led to a corresponding increase in waste generation [8, 9].

In 2018, according to the Food and Agriculture Organization [4], the global production of wood waste surpassed an impressive figure of 234.6 million cubic meters. China undoubtedly stands as the preeminent producer of this particular waste, commanding a remarkable 44.3% share of the global production [4]. Following in its wake, Brazil contributed a noteworthy 8.2%, and the United States made a commendable contribution of 6.1% [4]. These staggering figures result from complex wood waste generation pathways that contribute to accumulating residual lignocellulosic biomass worldwide.

One of the most significant sources of wood waste is logging activities and wood processing, particularly in countries with large forest resources and robust wood processing industries, such as China, Brazil, and the United States. During logging operations, residues such as branches, treetops, and stumps are often left behind, leading to a considerable amount of unutilized biomass [10]. Sawmills also generate substantial quantities of wood waste, including bark, sawdust, and wood chips, which are by-products of the lumber production process [11]. Another pathway that contributes to wood waste generation is the abandonment of forests. This often occurs due to shifts in land use priorities or economic factors, leading to the neglect and mismanagement of forest resources [12]. As a result, unmanaged or abandoned forests experience increased dead wood accumulation, as fallen trees and branches are left to decompose on the forest floor [13]. This not only represents a loss of valuable biomass but also poses potential risks, such as increased wildfire hazards. Forest fires are a significant contributor to wood waste generation, and their frequency and intensity have been exacerbated by climate change [14]. The aftermath of a wildfire leaves behind substantial amounts of burned wood and charcoal, which can be considered a form of wood waste [15]. Managing and utilizing this fire-affected biomass presents unique challenges and opportunities for sustainable wood waste reuse strategies. Natural disasters, such as storms, hurricanes, and other extreme weather events, also generate wood waste. These events can cause widespread tree damage, uprooting, and breakage, resulting in large volumes of wood debris scattered across affected areas [16]. Additionally, insect outbreaks and disease infestations can lead to tree mortality, further contributing to the accumulation of wood waste in forests [17]. As a result, there is a growing need to find sustainable solutions for managing and utilizing these substantial quantities of wood waste.

The utilization of wood waste has gained significant attention in recent years, and wood classification systems, such as the German legislation Altholz V from 2012, play a crucial role in optimizing its management [18]. The Altholz V system categorizes post-consumer wood waste into four distinct quality grades: Q1, Q2, Q3, and Q4, based on their properties, characteristics, and potential for recycling or disposal [18]. Q1 represents untreated wood or wood with negligible contamination and is highly suitable for recycling. Q2 consists of treated wood without halogenated organic compounds in the coating and is moderately suitable for recycling [18]. Q3 includes treated wood with halogenated organic compounds in the coating and has low suitability for recycling, generally destined for incineration [18]. Q4 represents wood waste impregnated with preservatives, which is considered hazardous waste and must be disposed of in specialized facilities [18]. This classification system promotes the efficient utilization of wood resources through the concept of "cascading use of wood" or "wood cascading," which refers to using wood resources in products that create the most economic value over multiple lifetimes [19]. The cascading use of wood involves prioritizing value-adding and resource-efficient wood applications before eventual energy recovery at the end of life [20]. This approach enables the conversion of abundant wood waste resources into various valuable products, thereby maximizing the economic and environmental benefits of wood waste utilization [20]. One of the most promising ways to utilize wood waste is through pyrolysis, a thermochemical process that converts wood waste into bio-oil, biochar, and syngas [21]. Another approach to utilizing wood waste is producing wood pellets, which can be used as a renewable energy source for heat and power generation [4]. In addition, wood fibers can be used to produce packaging materials, such as biodegradable plastics and paper products. Moreover, wood waste can be used in agriculture as a soil amendment to improve soil fertility and structure [22]. Wood ash, a by-product of wood combustion, contains nutrients such as potassium and calcium that can benefit plant growth [23].

The existing body of literature primarily focuses on various sustainable approaches to harnessing the potential of wood waste. However, the concept of sustainability is often not precisely defined, and the trade-offs between environmental, economic, and social objectives remain underexplored. This gap in the literature is substantial because it hinders stakeholders from effectively distinguishing between practices that genuinely promote responsible resource utilization and those that inadvertently perpetuate ecological, social, and financial challenges. Consequently, this review addresses this gap by evaluating practices for utilizing wood waste through ecological, economic, and social lenses. By systematically assessing which practices are sustainable and which are not, this study will offer a comprehensive framework for distinguishing between strategies aligned with sustainability principles and those that may unintentionally perpetuate unsustainable resource utilization. The outcomes of this review will equip researchers, industries, policymakers, and environmental advocates with the necessary knowledge to make informed decisions that genuinely contribute to the sustainable utilization of wood waste.

## Methods

### Protocol

This scoping review protocol was developed using the methodological framework outlined by Arksey and O’Malley [24]. A priori protocol guides the stages of the scoping review, from research question formulation to identifying relevant literature, study selection, data extraction, quality appraisal, and synthesis. The draft protocol was revised through an iterative process based on feedback from the multidisciplinary research team consisting of sustainability issues experts and synthesis methodology knowledge. This input clarified concepts related to wood waste streams, reuse applications, and sustainability impacts while ensuring methodological rigour. The final protocol provides a comprehensive plan for executing the scoping review, maximizing scientific quality and consistency.

### Eligibility criteria

This scoping review considered a broad range of literature on the sustainability of reusing wood waste across different applications. This review included primary research studies, books, and reviews published in English. The definition of wood waste encompasses various residual lignocellulosic biomaterials originating from forestry, wood processing, construction/demolition activities, and post-consumer discarded wood products [25]. Specific wood waste streams of interest include, but are not limited to, sawdust, wood chips, bark, logging slash, recycled lumber, paper fibers, demolition debris, and wood-rich municipal solid waste [25].

All types of reuse pathways for these waste materials were eligible, including energy generation, manufacture of wood products like particleboard, mulching and landscaping, agricultural applications, civil construction, preservative treatments, and other industrial uses. This review examined the sustainability of wood waste reuse across environmental, economic, and social dimensions. As such, studies investigating relevant technical, ecological, health, environmental, business, and social factors related to the sustainability of reusing wood waste materials were included. This review excluded studies focused solely on forestry practices for virgin timber rather than waste wood reuse. Animal studies and non-English publications were also excluded based on relevance and feasibility considerations. After preliminary searching, eligibility criteria were refined further to demarcate relevant literature. Clear specification of wood waste sources of interest, reuse applications, and sustainability perspectives ensured the review captured publications most relevant to the research objectives.

### Information sources and search strategy

Comprehensive literature searches for potentially relevant studies were conducted using the following multidisciplinary databases: Scopus, Web of Science Core Collection, ScienceDirect, and Google Scholar. These databases provided extensive coverage of literature on forestry, agriculture, environmental science, sustainability, and allied fields where studies on wood waste reuse applications were published. The search combined controlled vocabulary terms as well as keywords in the title, abstract, and keyword fields for concepts relevant to wood waste, reuse categories of interest, sustainability impacts, and other pertinent topics. Reference lists of all included articles were hand-searched to identify further relevant citations. During the searches, no limitations were imposed on publication year, language, or geographic region. Preliminary searches indicated a large volume of potentially pertinent literature, so date or language restrictions are not required for feasibility. However, eligibility criteria were narrowed during study selection after assessing the scope of initial results. Comprehensive searches without restriction were planned to identify the breadth of available literature on sustainable wood waste reuse.

### Data extraction

Data extraction was conducted using standardized forms to collect key details from included studies in a consistent manner. A draft extraction form was piloted on a random subset of 5 included articles to refine categories and ensure comprehensiveness and usability. The final form extracted relevant bibliographic details, wood waste materials, reuse purpose, sustainability factors analyzed, methodologies, and results/conclusions for quantitative and qualitative synthesis. Formal quality appraisal of individual studies was not undertaken, which was consistent with the scoping review methodology. However, basic study details and limitations were recorded. After sufficient reliability was confirmed during piloting, data extraction on all included studies was initially performed. This data was verified and extracted to validate accuracy and completeness. Revisions were checked to ensure integrity. This approach was to minimize error and omission.

### Methodological appraisal

In keeping with the accepted scoping review methodology, no formal quality appraisal or risk of bias assessment was conducted for individual studies included. This diverges from systematic reviews, where study quality assessment is a key component. However, as scoping reviews aim to present a broad mapping of available evidence on a topic irrespective of quality, such appraisal was generally not undertaken. This precludes excluding papers with a higher risk of bias or lower quality while maintaining feasibility for the broad scope. However, highly low-quality studies were excluded if they did not meet minimum standards for inclusion. Fundamental methodological details and limitations were recorded during data extraction to provide context on evidence provenance without formally scoring quality. This approach enabled a comprehensive synthesis of the available literature while acknowledging study rigour and bias variability.

### Literature search

A comprehensive literature search was conducted to identify studies on the sustainable and unsustainable reuse of wood waste. The following databases were searched from inception through February 2023: Scopus, Web of Science Core Collection, and Google Scholar. Targeted searches combined relevant keywords such as "wood waste", "reuse", "sustainability", and terms for specific applications like "renewable energy", "mulch", and "preservatives". Grey literature was sought through Google Scholar. Reference lists of included studies were also hand-searched. No geographic or language limits were applied.

The first step in the study identification process was comprehensive searches across relevant academic databases. These initial searches yielded a total of 1,150 records. A deduplication process was undertaken before formal screening procedures began, identifying and removing any duplicate records among the 1,150. This deduplication resulted in the removal of 350 records, leaving 800 unique records from the databases to move forward into the screening phase.The 800 records underwent a preliminary screening based on predefined eligibility criteria for the review. This initial screening process aimed to efficiently filter the records down to those appearing most relevant to the research topic and question. By applying the eligibility criteria, 425 records were excluded, and the results were narrowed down to 375 records, which warranted seeking full-text reports for further assessment. The 375 full-text reports were then requested and sought for retrieval from the various journals, publications, and sources. However, 74 of the reports could not be successfully retrieved despite the efforts to obtain the full text. Therefore, 301 full-text reports could be assessed for eligibility through full-text review.

**Fig 1:**
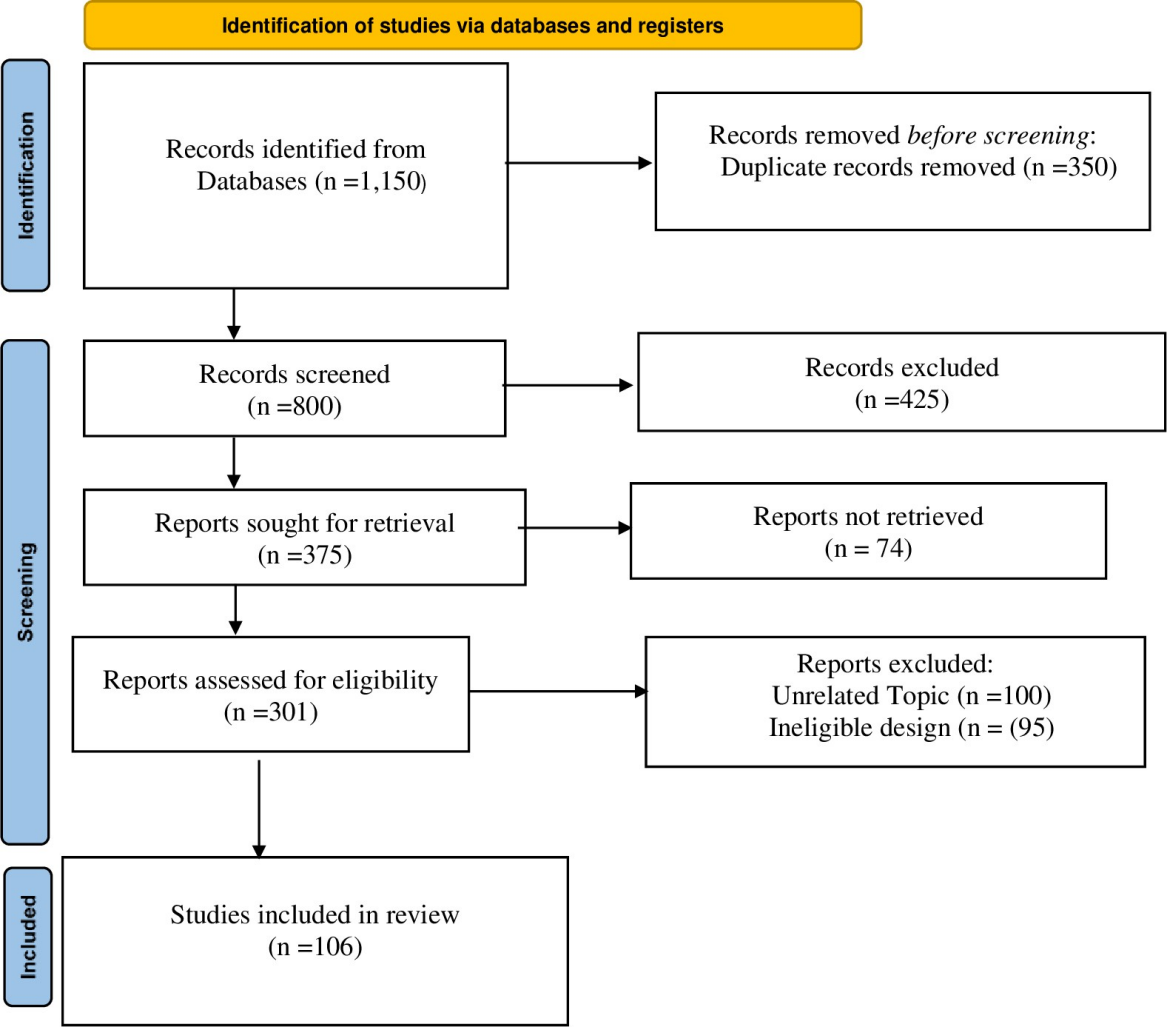
Prisma flow diagram.

This subsequent round of in-depth eligibility screening at the full-text level resulted in 100 studies being excluded for covering unrelated topics outside the scope of inquiry and 95 studies being excluded for employing ineligible study designs unfit to appropriately address the stated research question. Following these comprehensive identification procedures and step-wise phases of screening and eligibility assessment, 106 studies satisfied all conditions and eligibility criteria to be included in the final review synthesis. Fig 1 show a Prisma Flow of the study, revealing included and excluded studies.

**Fig 2:**
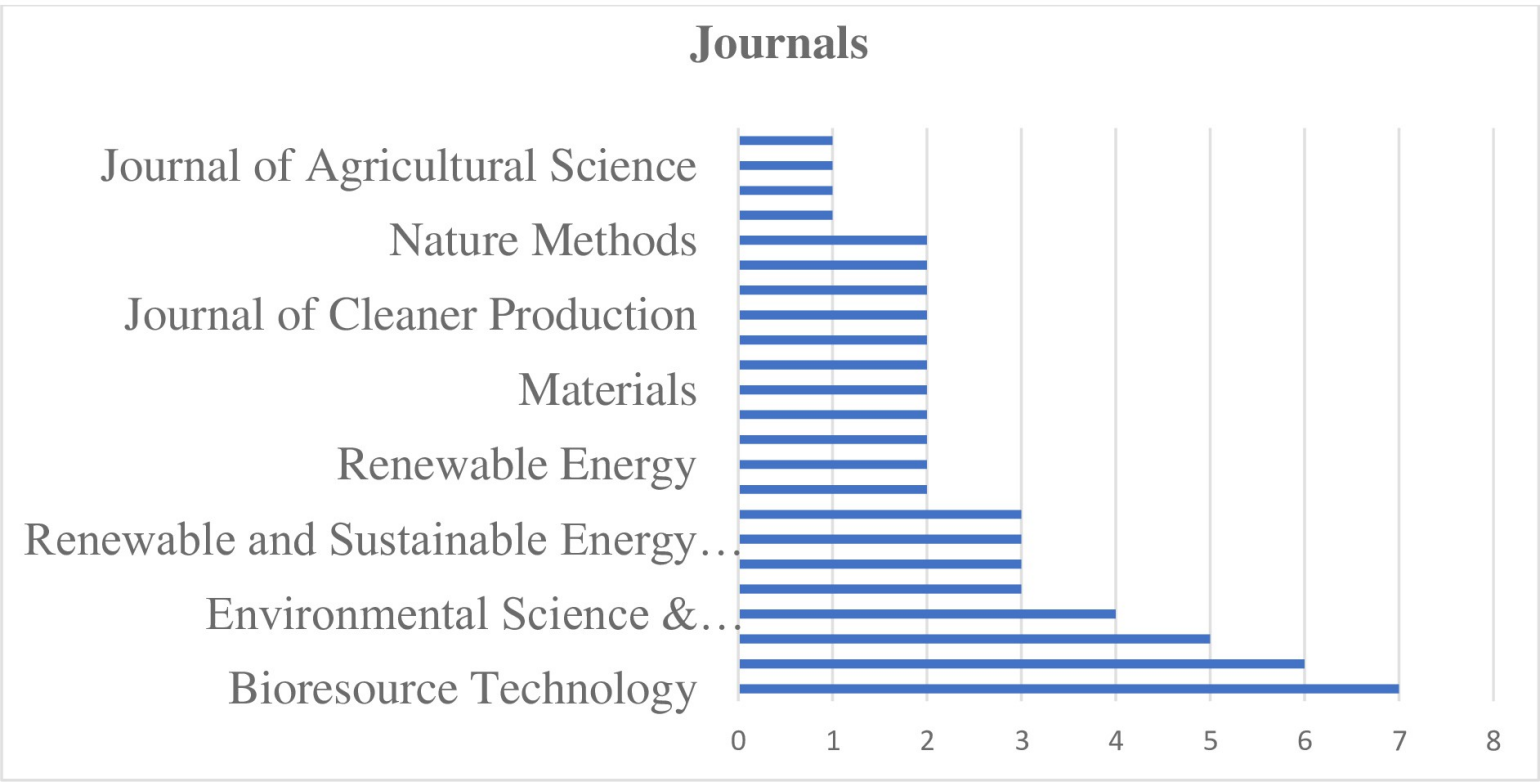
Wood waste utilization research: Publications across journals.

Fig 2 presents the frequency of publications on wood waste utilization research across various scientific journals. The analysis reveals that Bioresource Technology is the most prominent journal in this field, with 7 publications, followed closely by Waste Management with 6 publications. Forest Ecology and Management rounds out the top three with 5 publications. This suggests that these three journals are key platforms for disseminating research in wood waste utilization. Environmental Science & Technology also shows significant contribution with 4 publications. Following this, there’s a group of journals each with 3 publications: Critical Reviews in Environmental Science and Technology, Applied Energy, Renewable and Sustainable Energy Reviews, and Chemical Engineering Journal. This indicates a broad interest in the topic across environmental science, energy, and chemical engineering fields. The data also shows a considerable number of journals (9 in total) with 2 publications each, including Applied Soil Ecology, Renewable Energy, Environmental Pollution, and Journal of Cleaner Production, among others. This suggests a wide-ranging impact of wood waste utilization research across various scientific disciplines. Interestingly, the majority of journals (38 in total) have only 1 publication each. This extensive list includes diverse fields such as agricultural science, atmospheric environment, energy policy, materials science, and even probiotics and animal sciences. The wide array of journals with single publications indicates the interdisciplinary nature of wood waste utilization research and its relevance to numerous scientific and technological domains. The distribution of publications across journals suggests that while there are a few key journals leading the field, wood waste utilization research is broadly disseminated across a diverse range of scientific publications. This reflects the multifaceted nature of the research, encompassing aspects of environmental science, waste management, forestry, energy, chemistry, and materials science, among others.

**Fig 3:**
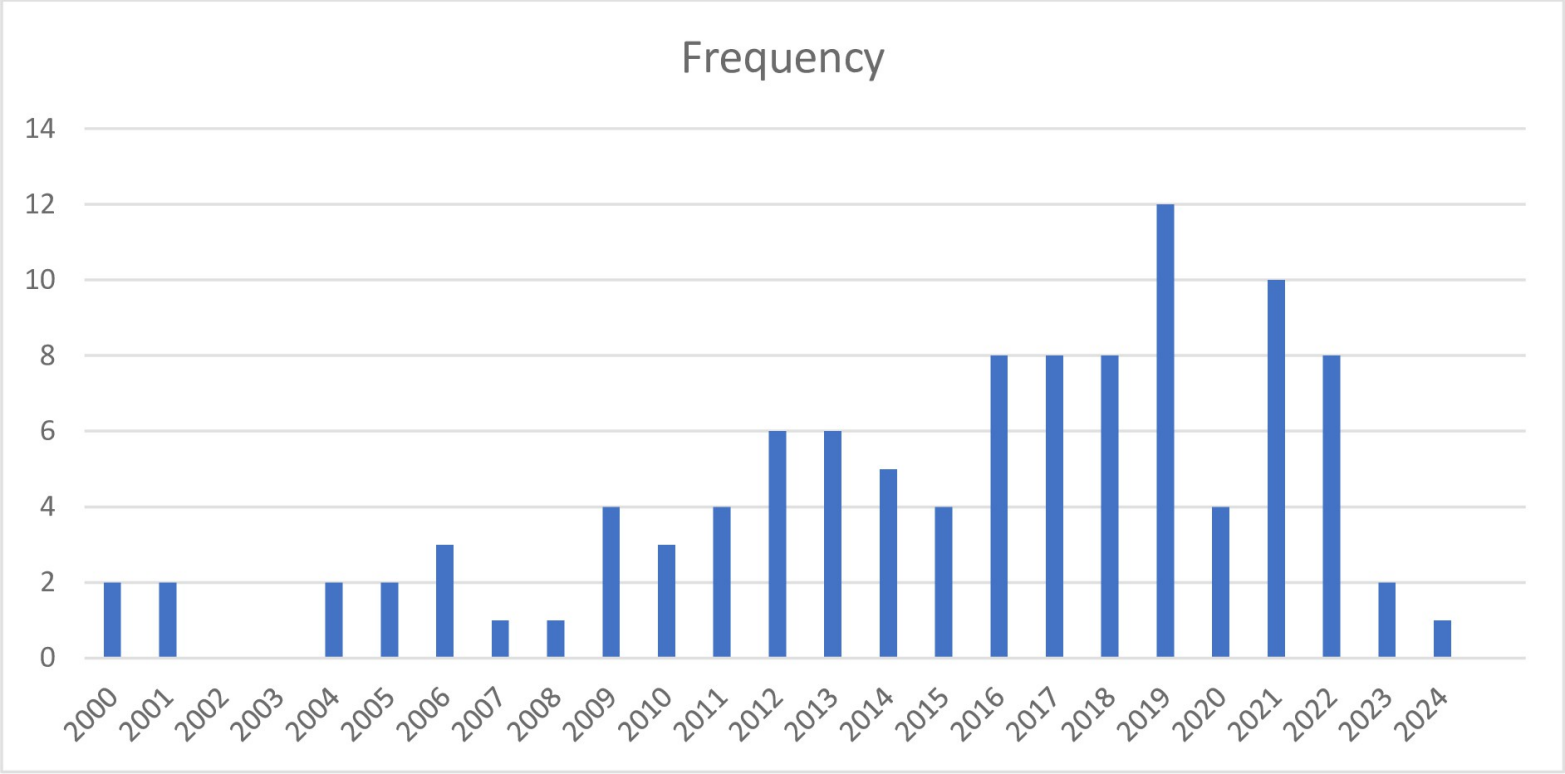
Yearly distribution of wood waste utilization research publications.

Fig 3 above presents the number of publications on wood waste utilization research from 2000 to 2024. The data reveals that the most active years for research in this field were 2021 (14 publications) and 2019 (12 publications), followed by 2017 (10 publications). The chart also shows that the years 2016, 2018, and 2022 each contributed 8 publications. This suggests a peak in research activity during the late 2010s and early 2020s. The years 2013, 2014, and 2015 saw a steady output of research, with 6, 6, and 5 publications, respectively. This indicates a consistent interest in wood waste utilization during this period. In the earlier years, 2009, 2010, and 2011 each had 4 publications, while 2005 and 2006 had 3 publications each. The early 2000s (2000–2004, 2007, 2008) and the most recent years (2023 and 2024) had the lowest number of publications, with only 1 or 2 per year. The distribution of publications across the years indicates that research on wood waste utilization experienced a significant increase between 2013 and 2022, with a notable contribution from 2013 to 2015. This trend suggests growing interest and investment in wood waste utilization research over the past decade.

### Synthesis

Data synthesis comprised quantitative and qualitative techniques aligned with the scoping review methodology proposed by [26]. The basic quantitative analysis summarized the types of wood waste materials, reuse applications, sustainability factors, and other key characteristics reported across included studies using frequencies and percentages. This descriptive numerical analysis elucidated the breadth of available evidence. Content analysis was then inductively categorized and synthesized text segments related to environmental, economic, and social dimensions of wood waste reuse sustainability. The data were coded and organized into thematic categories using NVivo qualitative data analysis software. Coding will be compared between reviewers to refine category definitions and conceptual linkages in the analytical framework through discussion. Quantitative and qualitative synthesis elucidated the current state and structure of research activity on sustainable wood waste reuse to map knowledge gaps and implications.

### Findings

This section presents findings on the literature’s sustainable and unsustainable wood waste reuse methods. This section presents findings on the sustainable reuse of wood waste. This was followed by the unsustainable reuse of wood waste in the literature.

### Sustainable use of wood waste

A thorough literature review indicated that the sustainable reuse of waste was done using *renewable energy*, *adsorbent*, *building construction*, *and fertilizer*.

### Renewable energy

Wood waste is a promising renewable energy source that can help address climate change and energy security concerns. Using various wood waste streams for energy generation through different conversion pathways, we can significantly reduce greenhouse gas emissions and fossil fuel consumption compared to conventional energy sources [27]. Moreover, using wood waste for energy production can play a crucial role in achieving a low-carbon energy transition while minimizing land use competition and biodiversity impacts [28]. Recent studies have demonstrated the feasibility, sustainability, and benefits of using various wood waste streams for energy generation through different conversion pathways.

One such conversion pathway is direct combustion, a well-established and effective method for generating renewable heat and power. Wood waste is an abundant, low-cost, and renewable fuel source that can be used for energy production with proper technology adaptations [29]. Increased utilization of wood waste reduces reliance on fossil fuels, lowers carbon and particulate emissions compared to coal, supports local economies, and provides a productive waste management solution [30]. Displacing natural gas with forest residuals for boiler heat energy in a lumber mill lowered net carbon emissions by 66% [30]. Co-firing wood waste with coal improved fly ash stability and lowered metal leaching [29]. At the same time, ongoing research efforts aim to limit particulate, NOx, and SOx emissions through advanced combustion control and flue gas treatment. A study by Carvalho et al. [31] investigated the co-firing of wood waste with coal in a fluidized bed combustor. The results demonstrated that co-firing up to 50% wood waste could maintain stable combustion conditions while reducing NOx and SO2 emissions. Additionally, Wang [32] evaluated the performance of a novel wood waste-fired boiler system with integrated emission control technologies. The study found that the optimized system could achieve high thermal efficiency and significantly reduce particulate matter, NOx, and SO2 emissions compared to conventional wood-fired boilers. A recent study Mohaghegh (2021) explored the use of wood waste in a novel hybrid solar-biomass power generation system, demonstrating improved overall efficiency and reduced emissions compared to standalone biomass combustion systems.

**Fig 4:**
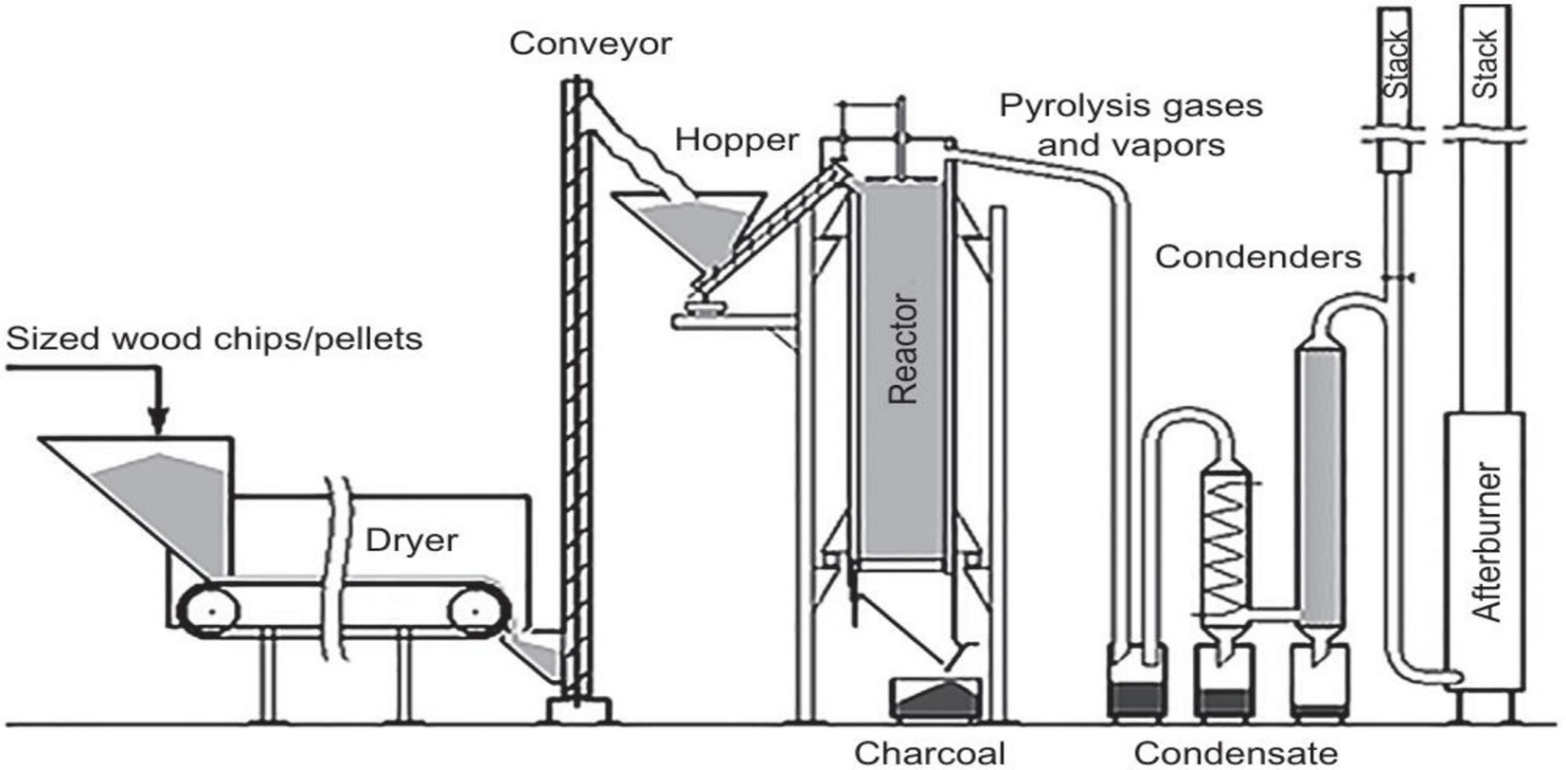
Wood pyrolysis [39].

In addition to direct combustion, pyrolysis and gasification are thermochemical conversion processes that transform wood waste into valuable biofuels and chemicals [33]. An on-site wood gasification system reduced greenhouse gas emissions by 70–180% compared to fossil fuel-dependent power supply, with a payback period of 2–4 years and 20% savings in heating costs over 20 years [34]. An innovative fast pyrolysis method aided by nano-catalysts resulted in increased bio-oil yields and tailored composition, demonstrating the promise of catalytic pyrolysis for selectively producing biofuels from low-value wood waste [35]. A study by Carpenter et al. [36] evaluated the techno-economic feasibility of a mobile pyrolysis system for converting forest residues into bio-oil. The results showed that the system could be economically viable and provide a sustainable way to valorize wood waste. Furthermore, Patel, Zhang and Kumar [37] investigated the co-pyrolysis of wood waste and plastic waste to produce high-quality bio-oil. The study demonstrated that co-pyrolysis could enhance the bio-oil yield and quality while offering a solution for the simultaneous management of wood and plastic waste streams. A recent study by Suriapparao and Vunu [38] explored the use of microwave-assisted pyrolysis for wood waste conversion, demonstrating improved energy efficiency and product yields compared to conventional pyrolysis methods. Fig 4 shows wood pyrolysis.

Apart from direct combustion, pyrolysis, and gasification, wood waste can also be densified into pellets, providing a convenient and standardized fuel for various applications. The excellent durability, high energy content, and reduced emissions of densified fuel briquettes manufactured from recycled glued wood waste have been confirmed [40]. Furthermore, pyrolysis of wood waste can produce biochar, a carbon-rich solid material with various applications, including soil amendment and carbon sequestration. The existing body of technical, economic, and environmental analyses provides robust evidence for the viability and advantages of utilizing wood waste for biochar production. Realized benefits include climate change mitigation, reduced fossil resource dependence, waste management solutions, and rural development opportunities [41]. However, careful selection of suitable conversion pathways and system configurations is needed to maximize sustainability, and continued R&D addressing efficiency, cost, and emissions will further enhance the prospects for the broad adoption of wood waste biochar systems [42]. A study by Sahoo et al. [43] investigated the production of biochar from wood waste and its application as a soil amendment. The results showed that wood waste biochar could enhance soil fertility, increase crop yields, and sequester carbon in the soil. In another study Tack and Egen [44] assessed the potential of wood waste biochar for the remediation of heavy metal-contaminated soil. The authors found that wood waste biochar could effectively immobilize heavy metals in the soil, reducing their bioavailability and uptake by plants. A recent study by Olugbenga et al. [45] explored the use of wood waste-derived biochar for wastewater treatment, demonstrating its effectiveness in removing organic pollutants and heavy metals from industrial effluents.

**Fig 5:**
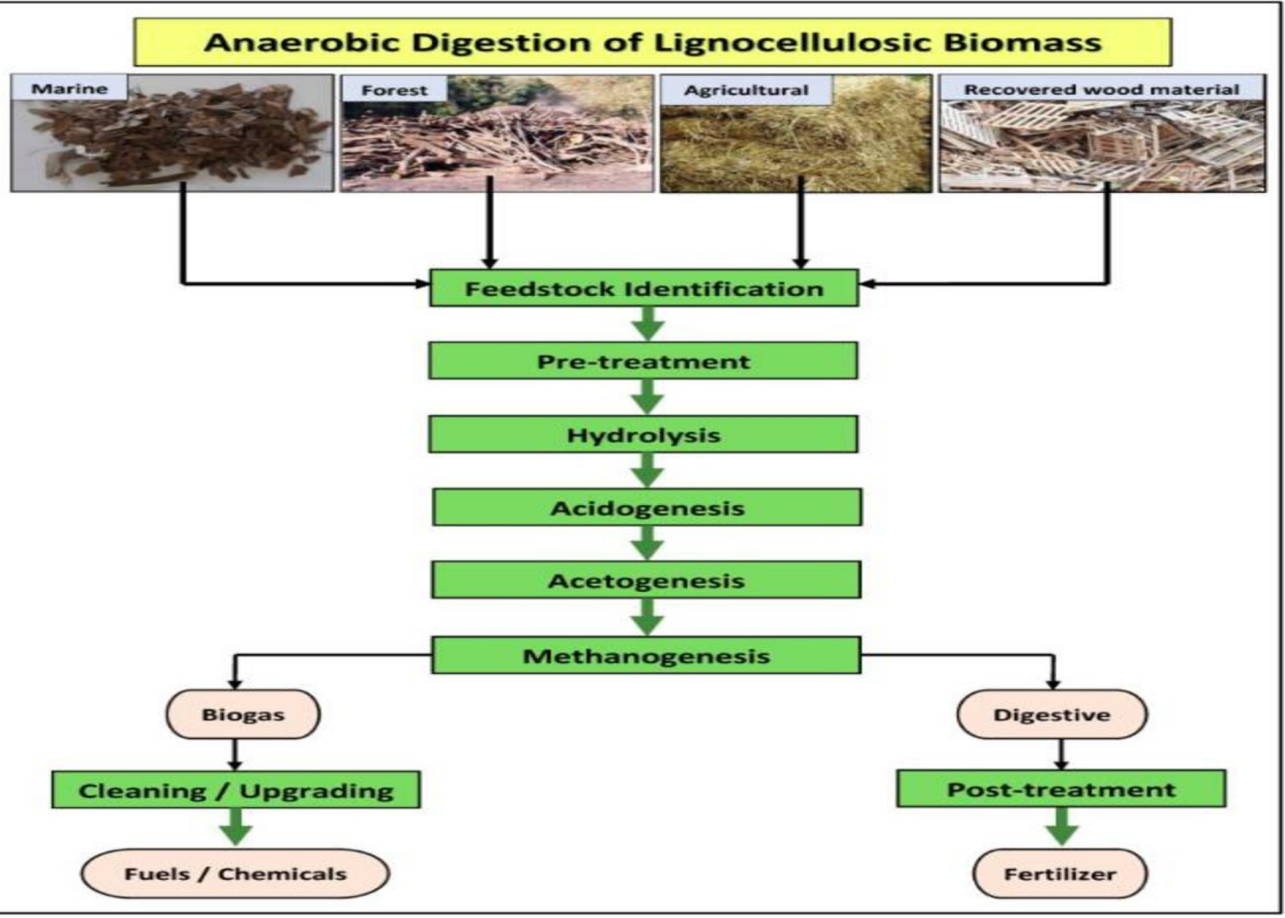
Anaerobic digestion of wood waste [51].

Furthermore, anaerobic digestion of wood waste can produce biogas, a renewable fuel primarily made of methane. Wood residues from urban tree species in Brazil were found to have high calorific values and energy density, making them a promising feedstock for biogas production [33]. Combined heat and power (CHP) systems using wood waste can efficiently generate electricity and proper heat. Life cycle assessments provide insights into the environmental performance of wood waste CHP systems [46, 47]. While wood waste use generally reduces fossil energy consumption and overall emissions, the magnitude of benefit depends on the reference system displaced and the specific conversion pathways and system configurations used [46, 47]. A study by Gokcol et al. [48] assessed the environmental and economic performance of a wood waste-based CHP system in Italy. The results indicated that the system could significantly reduce greenhouse gas emissions and energy costs compared to fossil fuel-based systems. Additionally, Madadian and Simakov [49] investigated the potential of integrating anaerobic digestion with pyrolysis to co-treatment wood waste and sewage sludge. The study found that the integrated system could enhance biogas production and energy recovery while producing valuable biochar as a by-product. A recent study by Gupta et al. [50] explored the use of artificial intelligence and machine learning algorithms to optimize the performance of wood waste-based CHP systems, indicating significant improvements in energy efficiency and emission reduction. Fig 5 reveals anaerobic digestion of wood waste.

### Adsorbent

As industrialization and consumerism increase, the management of solid wastes presents more significant challenges on a global scale. Nevertheless, waste biomass originating from wood processing and forestry operations is an abundant yet underutilized resource. Recent research has provided evidence of the considerable potential for wood waste to be utilized sustainably as an adsorbent in industrial and environmental remediation processes.

**Fig 6:**
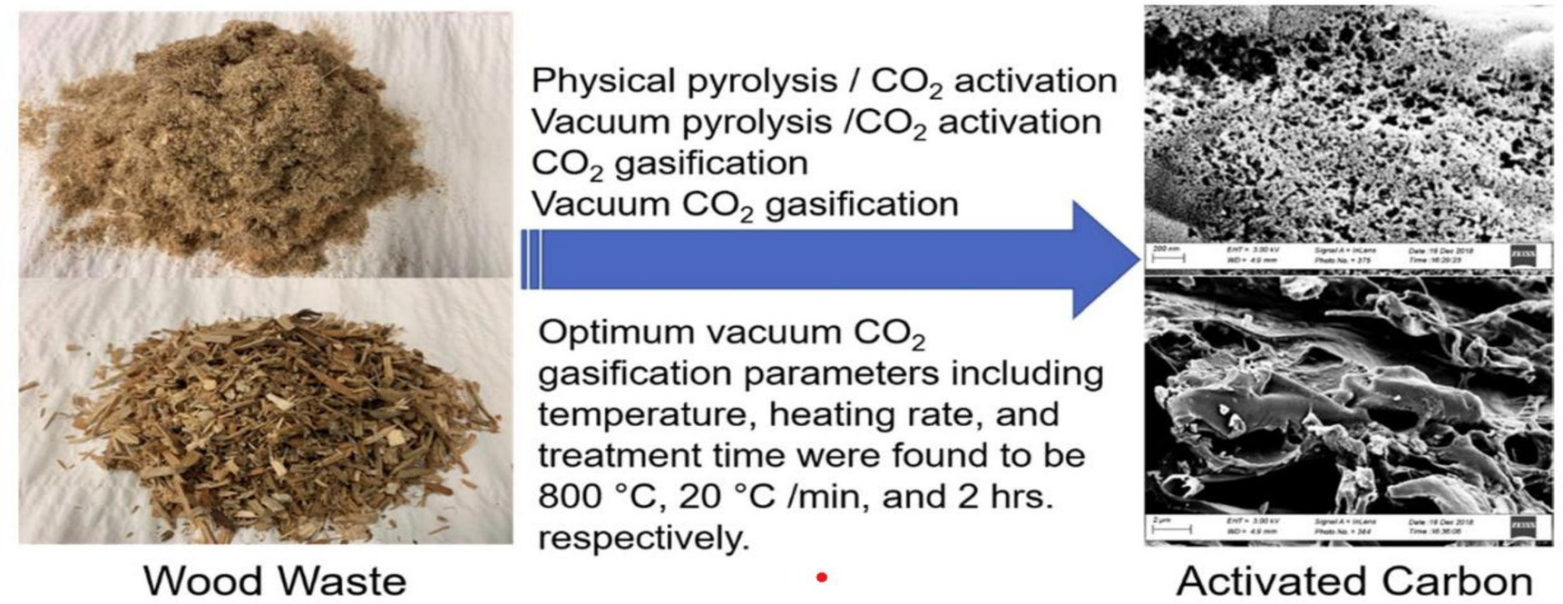
Activated carbon adsorbents [62].

Recent studies reveal strong potential to convert these residual biomass streams into activated carbon adsorbents via thermal processing and activation [52]. Compared to conventional activated carbons derived from fossil fuel precursors like coal, biochar from low-cost wood wastes can exhibit comparable or superior adsorption capacity for heavy metals, dyes, pharmaceuticals, and other priority pollutants in water treatment [52–55]. The excellent performance stems from the high surface area, tuned surface chemistry, and porous structure imparted during pyrolytic carbonization and physical/chemical activation [56, 57]. The biomass feedstock properties, production conditions, and activation processes strongly influence the characteristics and adsorption behaviour of the resulting bio-char adsorbents [52, 58, 59]. Steam activation, phosphoric acid activation, carbon dioxide activation, and nitric acid activation are among the processes that enhance the development of porosity and active adsorption sites in wood-based bio-chars [53, 54, 60]. For example, H3PO4-activated pine sawdust bio-char achieved a methylene blue adsorption capacity of 305 mg/g [32]. CO2-activated forestry waste adsorbed up to 288 mg/g of Pb (II) ions [57]. Optimization of production conditions can maximize economic feasibility while achieving high adsorptive performance. Januszewicz et al. [61] prepared activated carbons from wood and straw wastes using both physical and chemical activation after pyrolysis. Based on adsorption evaluations, the chemically activated carbons had higher surface area and showed potential for cleaning real industrial wastewater [61]. This study demonstrates the essential role of activation techniques in developing porous adsorbents from waste wood [61]. Fig 6 shows wood planes from activated carbon adsorbents.

Beyond activated carbon adsorbents, continuous column trials have shown effective removal of gas and liquid pollutants using wood-derived bio-char adsorbents, confirming their promise for scaled-up environmental applications. Over 90% of chromium (VI) removal from synthetic wastewater was demonstrated using bio-char produced from Melia azedarach wood waste [54]. Over 80% of copper and cadmium removal from electroplating wastewater effluents was obtained using pecan shell-based bio-char columns [63]. Removal of 65–85% of phosphate, nitrate, and organic matter from agricultural drainage using wood bio-char filtration systems has been reported [57]. Bio-char adsorption columns also effectively captured CO2 from flue gas streams, achieving uptakes comparable to commercial zeolite adsorbents [10].

While promising, concerns remain regarding long-term stability, reuse, and contaminant leaching. Accelerated aging studies show moderate increases in nutrient leaching over time, although concentrations remained within acceptable limits for soil application [64, 65]. Microbial degradation and abrasion during reuse can degrade adsorption performance, necessitating the development of standard reactivation procedures [66]. There are also uncertainties regarding the impacts of mineral components and carbon loss during field-scale operations [65]. Proper selection of suitable feedstocks and responsible production conditions are vital to enhance adsorbent stability while preventing secondary pollution [52]. For example, higher pyrolysis temperatures strengthen bio-char stability by increasing fused aromatic structures but can reduce adsorption capacity [58]. Acid washing and separation steps can selectively remove problematic ash components [59]. Yi et al. [67] synthesized a magnetic biochar adsorbent from wood waste that rapidly adsorbed Rhodamine B dye, showing its potential for cleaning contaminated waters. Salamat et al. [68] characterized and tested untreated wood industry waste for adsorbing dyes from textile plant effluent. Owing to its native porous structure and surface functional groups, the waste wood exhibited decent adsorption capacity without extensive processing, indicating its intrinsic attributes may be leveraged for adsorption.

Lignin, a major component of wood and other plant materials, has been found to be a promising material for creating sustainable adsorbents. Recent studies have shown that lignin-based adsorbents can effectively remove various contaminants from water, including heavy metals, dyes, and organic pollutants [69]. By chemically modifying lignin, researchers can improve its ability to adsorb pollutants by increasing its surface area and the number of active sites where molecules can attach [32, 70, 71]. Additionally, lignin is biodegradable and can be obtained at a low cost, making it an environmentally friendly and economically attractive option compared to traditional adsorbents [72]. Researchers have also begun exploring the use of lignin-based adsorbents for capturing gases, such as carbon dioxide (CO2), which is a major contributor to climate change [73]. For example, a lignin-based adsorbent modified with polyethylenimine showed a high CO2 adsorption capacity and selectivity, making it suitable for post-combustion CO2 capture applications [74]. Similarly, lignin-based activated carbons prepared using different activation methods showed that the ZnCl2-activated carbon exhibited the highest CO2 adsorption capacity due to its well-developed microporous structure and high surface area [75]. These studies highlight the potential of lignin-based adsorbents to contribute to a circular economy by valorizing waste materials and addressing environmental challenges, such as water pollution and climate change.

### Construction

Rising volumes of wood waste from forestry, industry, and construction activities pose significant disposal challenges and offer opportunities for recycling lignocellulosic residuals into sustainable building materials [18, 76]. Recent studies have explored various valorisation pathways for diverting wood waste into components like concrete blocks, mortars, and particleboards.

Several investigations have focused on partially substituting conventional concrete ingredients with wood waste. Sawdust and wood scrap aggregates were used to replace cement, sand, and crushed rock at incremental ratios of 10–30% by volume. In a study conducted by Abed et al [77], after 28 days of curing, compressive strength testing showed that 20% substitution across all ingredients produced concrete blocks with adequate structural strength, meeting ASTM C90 standards [77]. Further increasing wood waste ratios progressively reduced compressive strength due to dilution effects but enabled lightweight blocks with density reductions up to 36% [77]. The study concluded that up to 20% wood waste incorporation can be cost-effective and environmentally friendly in concrete block design [77]. Expanding beyond blocks, wood aggregate was assessed as a replacement for crushed granite in concrete mix designs [78]. With 15% coarse aggregate substitution, the resulting concrete showed 3.06% higher 28-day compressive strength (32.36 MPa) and 9.75% improved split tensile strength versus the control concrete [78]. Static modulus of elasticity and ultrasonic pulse velocity were also maintained [78]. The wood aggregate concrete had a 267% higher slump, overriding the water demand concerns of highly porous residues [78]. The performance enhancements were attributed to the lower density and better adhesion of wood particles [78]. At finer scales, waste wood powders and fibres were introduced as sand replacements in cement mortars, ranging from 10–50% by mass (Ince et al., 2021). Consistent with dilution effects, increasing wood content lowered compressive strength, flexural strength, and dynamic elastic modulus [79]. However, even at 50% levels, the strength retention was found adequate for non-structural building applications as per ASTM C270 standards [79]. Natural resources and emissions associated with virgin binder and aggregate production are conserved by regenerating wood waste into mortars. Beyond lab-scale studies, life cycle assessment models were used to compare the environmental profiles of four wood waste management scenarios [80]. While the current practices of landfilling and incineration had high impacts, manufacturing particleboards from wood waste lowered greenhouse gas emissions by 34–77% across impact categories [80]. However, utilization as biofuel feedstock for energy generation achieved the highest reductions [80]. The study concluded that cascading wood waste into construction materials and bioenergy applications is imperative to improve the sustainability of waste flows [80].

**Fig 7:**
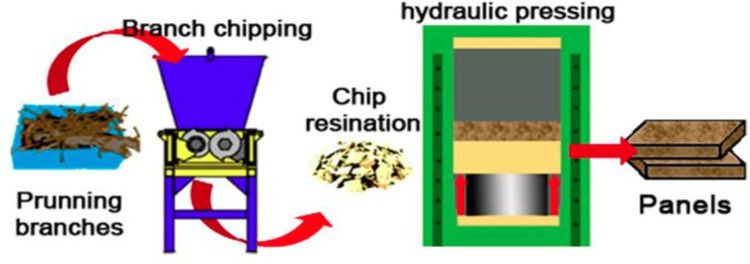
Wood planes from wood waste [88].

Particleboard manufacturing has attracted particular interest as a productive reuse pathway for recycled and residual wood. Increasing applications of wood waste in particleboards, moving beyond direct combustion or landfilling, have been reviewed [81]. Agricultural residues like straw, bamboo, and bagasse can replace virgin wood fibre, supported by studies on their mechanical suitability [81]. Oil palm biomass was found to produce boards meeting strength and dimensional stability requirements [82]. However, balancing realistic fractions with cost and quality control remains a key challenge [83]. Pine forest waste comprising bark, sawdust, and splinters was incorporated at 10% and 20% in particleboards [84]. While increasing waste wood lowered the modulus of rupture, tensile strength, and screw-holding capacity, the panels still met furniture grade standards [85]. Preconditioning the waste wood to lower moisture content was recommended to improve dimensional stability [85]. Particleboards were manufactured from recycled construction and demolition wood waste, including medium-density fibreboard, plywood, and timber scrap [82]. The panels met density, water absorption, swelling, and tensile strength benchmarks for interior-grade products, demonstrating waste-cascading potential [82]. Particleboards made from recycled wood were compared to fresh spruce boards [86]. The recycled wood boards showed improved dimensional stability and biological durability, though lower mechanical strength [86]. The study advocated for standards supporting higher recycled content in structural boards [86]. Underused non-forest biomass options have been reviewed, suggesting prioritization of wastes based on quantity and quality factors [87]. The development of flexible hybrid furnishes can balance waste utilization with end-use requirements [87]. Fig 7 shows wood planes from wood waste.

While wood particleboards have been extensively investigated, fewer studies have focused on fine wood waste reuse in materials like mortar and concrete. Wood processing residuals like sawdust and powders were incorporated in lightweight cement mortars, ranging from 2.5–10% by mass [89]. Compressive strength declined beyond 5% additions but remained structurally adequate [89]. Thermal conductivity was also reduced by 25% with wood waste inclusion [89]. The study recommended water reducing plasticizers to enable 10% substitution without drastic strength losses [89]. Coarse and fine sawdust were introduced to replace cement in self-compacting pastes partially [90]. The wood waste lowered density and shrinkage cracking by up to 80% compared to control pastes, acting as an internal curing agent [90]. However, sawdust also decreased compressive strength, restricting viable fractions to 5% in structural applications [90]. The presence of wood waste as either aggregates or fines tends to increase porosity and water demand while diluting the binder component, leading to trade-offs between sustainability goals and mechanical performance. Research continues to identify optimal substitution levels by balancing these factors through narrative reviews [83], quantitative syntheses [91], and statistical modeling [92]. The common theme across these studies is the significant potential to divert abundant, low-value wood waste from landfills and leverage the renewable properties of lignin and cellulose. Extensive lifecycle inventories for wood product manufacturing have been compiled, which can also guide waste-to-product processes [93]. Recycling wood waste directly displaces demand for virgin lumber, conservatively estimated at 1.1 green metric tons per 1 cubic meter of woo d by the US EPA. Cement and concrete production account for 7% of global CO2 emissions, incentivising wood substitution [94]. However, lignocellulosic residues pose unique challenges like low density, high moisture absorption, and dimensional stability concerns, which can undermine structural performance compared to inorganic ingredients [94]. Pre-processing via drying techniques helps address these limitations [94]. Chemical or thermal modification can also tailor the hydrophobicity, adhesion, and degradation resistance of recycled wood particles [95].

### Fertilizer

Wood waste, such as sawdust, wood chips, and other lignocellulosic materials, can be used as soil amendment and fertilizer. Several studies have explored the feasibility and effectiveness of using these waste materials in agricultural applications. The research suggests that wood wastes can improve certain soil properties and provide nutrients, especially over extended periods as the materials decompose. However, the effects seem to depend on the specific composition and properties of the wood waste used.

Several studies have tested wood waste as a soil amendment to improve soil structure and water retention. For instance, composted yard waste, including wood chips, increased moisture retention in sandy soils when applied at 25% and 50% by volume [96]. The compost also improved soil nutrient contents and supported microbial biomass and activity [96]. Based on pot experiments, sawdust amendment improved water retention and availability in sandy loam soil, with 10% sawdust increasing plant-available water by over 18% [97]. Relatedly, increased water-stable aggregation in silt loam soil was observed after red oak sawdust application, raising soil organic matter levels [98]. The evidence indicates that adding wood wastes like sawdust and chips can modify soil’s physical properties to retain more plant moisture and nutrition.

Wood ash from burning wood wastes also demonstrates the potential to alter soil conditions, largely by modifying pH. Multiple studies have observed that wood ash addition significantly raises pH in acidic soils [99]. For instance, a liming effect and pH increase of 1.5 units was recorded after ash application in acidic forest soils [100]. Solubilized nutrients like calcium and magnesium from ash likely contributed to the liming effect [99]. However, excessive applications of over 10 tonnes/ha have risked pH increases above ideal ranges along with salt buildups [99]. Proper wood ash reuse as a liming agent can help sustainably improve acidic soils. Wood ash demonstrates more instant impacts as a nutrient source equaling or exceeding conventional fertilizers. Comparable ryegrass yields were observed between wood ash and triple superphosphate treatments on acidic soils [99], while up to 50% higher grass yields were recorded with ash [101]. The ash provides readily soluble potassium, calcium, magnesium and phosphorus [102]. However, excessive salt concentrations from the over-application of pure ash can damage crops [99]. As Börjesson, Hansson and Berndes [103] indicates, wood ash shows promise as a recyclable fertilizer, but suitable application rates remain unclear. A study by Baloch [104] studies on wood ash application in agriculture found that, on average, crop yields increased when wood ash was applied, depending on soil type and crop species. In terms of providing nutrients directly to plants, several researchers have observed some positive effects from wood waste fertilizers, especially over longer time scales. For example, a mix of sawdust, wood ash, and limestone applied to nutrient-deficient soils increased maize growth and leaf nitrogen content after two years of application [105]. However, yields were still lower than those of conventional fertilizers [105]. The nutrients were likely released slowly through decomposition. Similarly, untreated oak sawdust application improved grape seedlings performance after 98 days, and it was proposed that the sawdust served as a slow-release source of nitrogen and other nutrients [106]. However, pure sawdust fertilization was detrimental to beans and maize growth compared to conventional fertilizers and composted sawdust, indicating fresh wood alone provides limited immediate nutrition [107]. Overall, the research indicates wood wastes like sawdust can improve soil fertility and give some plant nutrients, but the effects emerge over extended periods as the materials break down.

While raw wood wastes demonstrate some promise as fertilizers based on the above studies, research has more consistently shown improved effects from processing the materials into compost before soil application. Several studies have compared the fertilizer effects of raw wood wastes versus composted wood wastes. For instance, yard waste compost resulted in significantly higher tomato and pepper yields in sandy soils than equivalent raw yard waste amendments, which contained up to 50% wood chips and sawdust [108]. The composting process likely made more nutrients available in plants. Similarly, composted sawdust more effectively increased plant growth, yield, and nutrition in pot experiments than raw sawdust at the same application rates, attributed to higher nitrogen availability in the compost [97]. A recent study by Kumar [109] explored the use of vermicomposting to process wood waste, finding that vermicomposted wood chips resulted in a 30% increase in lettuce yields compared to raw wood chip applications, likely due to enhanced nutrient availability and improved soil structure. The consensus is that composting helps release and retain nutrients from wood wastes that crop plants can take up.

### Unsustainable

The review indicated that the unsustainable reuse of waste included wood preservatives, Energy and Fuel Generation, and mulching.

### Wood preservatives

Timber products treated with chemical preservatives have an extended lifespan compared to untreated wood. However, many commonly used wood preservatives like Chromated Copper Arsenate (CCA), pentachlorophenol (PCP) and creosote pose sustainability issues regarding toxicity and environmental persistence. Reusing treated timber waste often perpetuates unsustainable impacts through contamination of waste streams and hazardous exposures. The literature has extensively documented how some of these timbers have been preserved.

Chromated Copper Arsenate (CCA) been extensively used to pressure-treat wood products since the 1930s, imparting resistance to decay, fungi and insects [110]. The key metallic agents in CCA are arsenic, copper, and chromium, which raise environmental and health concerns due to their toxicity and bioaccumulation potential. Khan et al. [111] tested CCA-treated wood samples collected from residential demolition sites in Florida for total arsenic and chromium content. Using a nitric acid digestion method, they found average arsenic and chromium retention levels of 1844 mg/kg and 1965 mg/kg, respectively, in the treated wood several decades after initial treatment. This indicates the persistence of these metals over the wood’s lifespan. Comparison with regulatory hazard limits showed that around 48% of samples exceeded the arsenic threshold for hazardous waste classification. Also, a study Moghaddam & Mullingan [112] examined the long-term leaching behavior of CCA-treated wood in landfill conditions. The results of the experiments showed that the pH of the leachants has a significant effect on the leaching process, and sulfuric acid (pH 3) is the most effective leachant compared to nitric and acetic acid. Hasan et al. [113] analyzed 38 different wood mulch products sourced from various retailers in Hawaii. Through nitric acid digestion and ICP-MS analysis, they discovered four samples (10–20%) contained CCA levels exceeding the voluntary standard of 66 mg/kg adopted by mulch industry associations. The presence of CCA-treated wood in municipal green waste collection and mulch production streams highlights contamination risks from reuse without proper sorting and diversion protocols. A follow-up study by Srivastava et al. [114] examined the effectiveness of various sorting techniques in reducing CCA contamination in wood mulch. They found that a combination of visual inspection scanning could reduce CCA contamination in mulch products. The leaching potential in CCA-treated timber reused in agriculture was studied by Veinott et al. [115]. They analyzed soil arsenic levels near CCA-treated wood stakes used to support grapevines in Florida citrus groves. Using graphite furnace atomic absorption spectroscopy, they recorded elevated soil arsenic concentrations averaging 49.4 mg/kg near the stakes, with the highest levels up to 98 mg/kg. Arsenic levels decreased rapidly with distance from the stakes, with no detection above the background beyond 1.2 meters. The leaching was likely accelerated under acidic soil conditions. Such studies confirm that CCA-treated wood can transfer toxic metals into surrounding soils, crops and waterways when reused, causing environmental contamination.

In addition to CCA, pentachlorophenol (PCP) has also faced usage restrictions over carcinogenicity and toxicity concerns but remains detectable in treated wood waste. Wiegand et al. [116] tested 127 recycled wood samples sourced from wood yards, landfills and other recycling outlets in the Portland, Oregon metropolitan area for PCP using gas chromatography. Around 20% of the samples contained PCP levels above the regulatory threshold of 50 ppm, making them unsuitable for unrestricted reuse. This pointed to PCP-treated wood not being adequately identified and diverted from municipal waste processing. Townsend and Solo-Gabriele [110] specifically examined PCP and arsenic content in used utility poles awaiting recycling in Belgium. Analysis using inductively coupled plasma mass spectrometry found average PCP concentrations between 139–241 mg/kg in the tested poles, with 16% exceeding the European hazardous waste limit of 500 mg/kg. The authors highlighted occupational health risks from exposure to PCP dust and vapor, especially during pole shredding and reprocessing of new wood products. Such evidence indicates potential PCP transfer and human exposure during reuse of treated wood waste.

Creosote-treated wood is also in the league of CCA and PCP. Utility poles, railroad ties and marine pilings are often treated with creosote to waterproof and protect the wood. Creosote is a complex mixture of over 10,000 compounds, predominantly polycyclic aromatic hydrocarbons (PAHs) [117]. Many creosote components are known carcinogens and toxicants. Kwon and Choi [118] investigated the reuse of creosote-treated railroad ties as landscaping timbers and fencing material at residential sites in Xi’an, China. Testing splinter samples via GC-MS detected 16 priority PAHs at levels ranging from 14 to 11,500 mg/kg, with a mean concentration of 2,300 mg/kg. Notable leaching into the soils below reused ties was also recorded, with mean total PAH measurements up to 4 times higher than controls [118]. Urine samples from residents near the reused ties showed elevated 1-hydroxypyrene, demonstrating exposure risks. The study evidenced that even relatively low residual creosote levels in reclaimed wood can mobilize and lead to contamination issues when reused [118]. Proper pre-treatment assessment is necessary to stem hazardous exposures through the waste stream [118].

The toxicity issues of conventional wood preservatives like CCA, PCP and creosote underline the need for more sustainable options with lower persistence and health risks [110]. Alternative preservatives such as copper azole, ammoniacal copper zinc arsenate (ACZA) and alkaline copper quaternary (ACQ) are touted to be more fixating and less leachable in wood while avoiding extremely toxic compounds [110]. Organic preservatives derived from plant extracts such as quaternary ammonia, furfural and essential oils have also shown promise with lower toxicity profiles than conventional options [119]. Wood modification techniques are also growing as preservative-free options, using processes like thermal treatment, acetylation and furfurylation to boost decay resistance [120]. Recent work by Klebert et al. [121] demonstrated the potential of plasma-assisted wood modification as a chemical-free alternative to traditional preservatives. Their study showed that plasma treatment could significantly improve the water repellency and decay resistance of wood without the use of toxic chemicals. Further assessment and adoption of alternative preservatives and wood protection methods can mitigate contamination concerns associated with reusing treated wood waste.

### Energy and fuel generation

Using wood waste as an industrial boiler fuel or feedstock for bioenergy provides an alternative to fossil fuels. However, researchers have identified sustainability issues with large-scale wood combustion related to emissions, harvesting impacts, and carbon balance. Several studies have revealed problematic air pollutants emitted when reusing wood waste as fuel. Sakai et al. [122] measured flue gas emissions from burning construction wood waste in small-scale boilers and found significant particulate matter (PM), carbon monoxide (CO), and volatile organic compounds (VOCs), including benzene, toluene, and xylenes. PM emission rates exceeded 2 g/kg wood while CO reached nearly 5 g/kg. Even with advanced control technologies, the authors noted potential health impacts from biomass boiler emissions. Similar concerns were raised by Bray et al. [123] in reviewing data on hazardous air pollutants (HAPs) from wood-fired boilers. Measurements showed high levels of formaldehyde, acrolein, and polycyclic aromatic hydrocarbons (PAHs), especially from the combustion of demolition waste wood, which often contains painted and treated material. Mean acrolein emissions of 9 mg/m3 substantially exceeded the safe Chronic Inhalation Reference Exposure Level of 0.02 mg/m3. The researchers concluded that wood waste boilers require stringent controls to limit public health risks.

Also, sourcing practices for wood fuel have sustainability implications. by removes nearly all above-ground biomass rather than just merchantable logs. While maximizing wood fuel supply, researchers have found WTH causes greater soil disturbance. [124] recorded 61% higher bulk density, 29% lower soil carbon, and 5–10 times greater erosion from WTH sites in spruce forests than conventional harvests. The impacts persisted for over 16 years after logging. At another WTH experiment site in Norway, Egnell [125] reported increased soil compaction, lowered macroporosity, and dramatically reduced soil-saturated hydraulic conductivity. WTH impacts on soil hydrology could negatively affect site productivity and revegetation [125]. The study emphasized better planning to minimize residual stand and soil damage from intensive biomass removal operations. A long-term study by Johnson et al. [126] examined the effects of WTH on soil nutrient dynamics and forest productivity over a 33-year period in mixed hardwood forests. They found that WTH sites showed a reduction in soil nitrogen and phosphorus levels compared to conventional harvesting sites, leading to decreased tree growth rates in subsequent rotations. Furthermore, a study conducted by Premer et al. [127] on WTH implications on forest growth and soil productivity. Their findings revealed that, on average, WTH resulted in a decrease in soil organic carbon, a reduction in soil nitrogen, and a decline in aboveground biomass production in the following rotation compared to conventional harvesting methods. Additionally, long-distance transportation of wood waste to supply centralized bioenergy facilities may also undermine sustainability. Dwivedi et al. [128] found that transport emissions largely offset the potential CO2 benefits of substituting wood pellets for coal at a power plant in Ontario when pellets were sourced from hundreds of kilometres away. Life cycle CO2 emissions increased between 68–94% depending on transport distance; similar results were reported by Sikkema et al. [129] for a US wood pellet facility relying on regional forest biomass. Moreover, Mitchell et al. [130] concluded that expanded biomass harvests for UK power plants would take over 100 years to achieve putative carbon savings when accounting for carbon pool impacts in the forest harvest areas [130]. A recent study by Kraxner et al. [131] explored global bioenergy scenarios and their implications for future forest development, land use, and trade-offs. The study highlighted the importance of considering spatial factors in bioenergy system optimization and demonstrated the potential for land-use conflicts and trade-offs between bioenergy production and other ecosystem services. The authors emphasized the need for integrated land-use planning and sustainable forest management practices to minimize negative impacts on forests and biodiversity. This finding has been corroborated and expanded upon by the seminal work of Searchinger et al. [132], which challenged the carbon neutrality assumptions of forest bioenergy and warned that Europe’s renewable energy directive could potentially harm global forests if not carefully implemented. The study argued that the directive’s incentives for wood-based bioenergy could lead to increased harvesting of forests, reducing their capacity to absorb and store carbon. The authors stressed the importance of considering the long-term carbon dynamics of forest bioenergy systems and called for a more nuanced approach to bioenergy policy that prioritizes the protection of forests and their role in mitigating climate change.

### Mulching

Reusing wood waste as mulch, landscaping features, and animal bedding provide environmental and economic benefits through waste diversion [133]. However, contamination and improper processing can make some reuse practices unsustainable. This review summarizes key studies on issues like nitrogen drawdown, salt toxicity, termite infestation, and metal contamination associated with wood-based mulching.

In terms of fresh wood mulch on soil nitrogen, uncomposed "fresh" wood mulch has exhibited detrimental impacts on plants’ and soil nitrogen availability. López et al. [134] investigated nitrogen immobilization from fresh sawdust and wood chip mulch application in Malaysian pineapple fields. Soil nitrogen levels decreased by over 90% in the first month after mulching with the nitrogen-poor materials [134]. This severely limited available nitrogen for plants, retarding pineapple growth and fruit yield compared to traditional practices using aged and composted mulch [134]. In pot experiments with basil, garden sage and tomato, Mitchell *et al*. [130] also recorded rapid soil nitrogen depletion and stunted growth with fresh wood chip mulch compared to composted mulch, with up to 68% lower biomass production. Immobilization lasts 1–2 months until the carbon in fresh wood mulch is partially degraded by soil microbes, after which nitrogen release occurs [134]. Proper ageing or composting of woody mulches is thus essential to avoid excessive nitrogen starvation [134].

Also, the potential salt toxicity has been well documented. Salt accumulation from wood-based mulches is another concern that researchers have studied. Whole-tree wood chips from disturbed roadside or construction site vegetation can contain high salt levels from soil contamination, potentially causing plant damage when used as mulch [135]. Benito et al. [136] recorded mulch electrical conductivity (EC, indicating salt content) ranging from 1.7 to 10 dS/m in a survey, with values above 2.5 dS/m considered risky. In experiments with landscape shrubs, mulches with EC greater than 3.0 dS/m increased soil salinity and reduced photinia and Indian hawthorn growth by over 40% [137]. Salt toxicity symptoms include leaf scorch, tip burn, stunted shoots and reduced root growth. To avoid plant loss, [137] advised testing wood chip mulch for salts and leaching with irrigation.

Reusing untreated wood waste as mulch has also been associated with increased termite activity and feeding damage. New [138] reported cases of Formosan subterranean termites inhabiting untreated cypress bark mulch used in commercial landscapes in Gainesville, Florida. Field samples showed that the termites readily consumed the wood particles. Replacing bark and wood mulches with inorganic materials eliminated termite presence and tree damage. Verma, Sharma and Prasad [119] also documented more significant subterranean termite counts in untreated wood/bark mulch compared to tree plots with rock or rubber mulch. Termites nesting in wood mulch can directly damage nearby trees by feeding on roots or structural weakness [119]. Pre-treatment of wood-based mulch using heat or natural extracts could help deter infestation.

Further, issues with metal contamination have been uncovered. Trace metals in reclaimed wood mulch are emerging as potential contaminants. Townsend et al. [139] detected elevated chromium, copper, nickel and zinc in mulches from recycled construction/demolition wood in the Eastern U.S. Metals likely originated from wood preservatives, paint and other material contamination. Metal levels exceeded ecological risk thresholds in 25% of samples [139]. High manganese and iron were also recorded, indicative of wood ash content. Bioassays was conducted by Guo, Liu and Zhang [140] showed reduced seed germination and plant growth in contaminated mulch, while leachate analysis revealed potential groundwater pollution risks. Guo, Liu and Zhang [140] emphasized the need for better waste wood screening and sorting to exclude painted/treated material from unrestricted mulch applications. In a related study, Gomes et al. [141] assessed the environmental risk associated with the use of waste wood as a landscape mulch. Gomes et al. [141] found that the presence of heavy metals in the mulch, particularly copper and chromium, posed significant ecological risks. The authors highlighted the need for sustainable management practices, such as source separation and contaminant removal, to ensure the safe use of waste wood mulch in landscaping applications [141]. These findings underscore the importance of adopting sustainable practices in the management of reclaimed wood waste to minimize environmental contamination and ensure the safe use of mulch in various application.

## Discussions

This review, which explores the utilization of wood waste, uncovers substantial potential benefits for sustainability and highlights notable limitations within current mainstream practices. Sustainable uses, including renewable energy generation, building materials, and soil amendments, offer a multifaceted approach to addressing economic, environmental, and social sustainability issues.

The economic relevance of wood waste utilization is particularly evident in reducing reliance on costly fossil fuels and providing a low-cost alternative for energy production [43], while stimulating rural economies through job creation and local industry support [142]. Establishing decentralized wood waste-to-energy facilities and developing local processing facilities for adsorbent production and building material manufacturing can create a wide range of employment opportunities, from low-skilled jobs in waste collection and transportation to high-skilled positions in processing and management. This economic diversification is particularly important for rural areas, which often struggle with limited job prospects and economic stagnation. As revealed by Dias [142], the positive impact of wood waste utilization on local industry and job creation provides further evidence for sustainable waste management’s role in promoting rural development. Ecologically, the importance of wood waste is emphasized by the significant potential of wood waste utilization in reducing greenhouse gas emissions. The use of wood waste for renewable energy production can lead to net emission reductions of up to 80% compared to fossil fuels [143]. This underscores the importance of transitioning to renewable energy sources to mitigate the impacts of climate change. Additionally, wood waste-based adsorbents and building materials have been shown to reduce pressure on landfills and minimize ecological impacts. This suggests that sustainable wood waste utilization can be crucial in promoting circular economy principles and reducing the environmental footprint of various industries [80]. The social benefits of wood waste utilization, as identified in this study, are particularly noteworthy. Community-based wood waste utilization projects can provide valuable capacity-building and knowledge-sharing opportunities, empowering local communities to take an active role in sustainable waste management [144]. The availability of low-cost, sustainable building materials derived from wood waste can also improve access to housing and infrastructure in developing regions, addressing critical social challenges. Moreover, the potential for wood waste-based adsorbents to enhance public health through water and air purification highlights the far-reaching social implications of sustainable waste management practices [145]. The importance of community involvement in wood waste utilization projects and the potential for these initiatives is to improve quality of life.

While some of the findings highlighted the numerous benefits of sustainable wood waste utilization, it is crucial to acknowledge that certain wood reuse practices raise significant sustainability concerns. This study has identified several practices, such as renewable energy generation, treated wood reuse, and mulching, that pose ecological, economic, and social challenges, undermining the overall sustainability of these practices. The ecological consequences of unsustainable wood waste management practices are particularly concerning. The findings of this study indicate that the combustion of wood waste for energy generation and intensive harvesting practices can lead to air pollution, soil degradation, and loss of biodiversity. These negative impacts can have long-lasting effects on the environment and the health of local communities, highlighting the need for more sustainable alternatives [146–148]. Additionally, reusing wood treated with toxic preservatives like CCA, PCP, and creosote has been shown to pose long-term environmental risks due to leaching and bioaccumulation [111]. This can lead to soil, water, and food contamination, further exacerbating the ecological damage caused by unsustainable wood waste management practices [149]. Moreover, the use of uncomposted wood waste mulch has been found to cause nitrogen immobilization, plant growth limitations, and reduced soil quality, as reported by López *et al*. [150] and Mitchell *et al*. [151], which can hinder the growth and productivity of agricultural systems [152]. The economic implications of unsustainable wood waste management practices are equally concerning. The findings of this study and that of Dwivedi *et al* [153] and Sikkema *et al* [154] suggest that the long-distance transportation of wood waste for bioenergy can be costly and offset potential CO2 benefits.

Furthermore, the carbon costs of increased wood extraction can undermine the financial viability of these projects [130]. These raise concerns about the economic sustainability of wood waste-based bioenergy initiatives and highlight the need for more efficient and localized solutions. Additionally, the high costs associated with disposing of or treating hazardous treated wood waste can make reuse projects economically unfeasible [111, 155]. Improper reuse can also lead to significant financial liabilities related to environmental contamination and human health impacts. These financial burdens can strain local communities and taxpayers, undermining the economic viability of unsustainable wood waste management practices. Moreover, trace metals in reclaimed wood mulch can result in costly remediation and increased operational costs, reducing the economic viability of mulching projects [156]. The social implications of unsustainable wood waste management practices, as identified in this study, are particularly alarming. Air pollutants from wood waste combustion and toxic compounds from reused treated wood have been shown to cause respiratory issues, cardiovascular problems, and increased cancer risk, particularly for communities living near industrial boilers, bioenergy facilities, or reuse sites [147, 148, 157, 158]. These raise serious public health concerns and highlight the need for stricter regulations and monitoring of wood waste utilization practices. Moreover, intensive harvesting practices for wood fuel supply have been found to negatively impact local ecosystems and communities, disproportionately affecting rural and indigenous populations [146, 159]. The loss of forest resources and ecosystem services can exacerbate social and economic inequalities, further marginalizing vulnerable communities. Additionally, the reuse of treated wood without proper precautions has been shown to perpetuate the use of toxic preservatives, prolonging exposure to hazardous substances and posing significant health risks to workers and local communities [160]. Furthermore, contaminated mulch can pollute groundwater [156], and termites in untreated mulch can damage buildings and trees, reducing the quality of life [138].

A comprehensive and holistic approach is necessary to address these unsustainable aspects of wood waste utilization. This should involve the development and implementation of policies and regulations that prioritize ecological sustainability, financial viability, and social well-being. Stricter emissions controls and monitoring should be enforced for wood waste-based energy generation, while sustainable harvesting practices that minimize ecological damage should be promoted. The use of eco-friendly wood preservatives and proper handling and disposal protocols for treated wood waste should be mandated to reduce environmental and health risks. Furthermore, it is crucial to engage local communities, stakeholders, and policymakers in the decision-making process related to wood waste management practices. Conducting comprehensive environmental and social impact assessments, ensuring transparency and public participation, and prioritizing the needs and concerns of affected communities are essential steps towards developing sustainable and equitable wood waste management strategies. Policies and regulations should incentivize sustainable practices, such as closed-loop systems, circular economy principles, and waste reduction and reuse while discouraging unsustainable practices that pose significant ecological, financial, and social risks.

## Conclusion

A review of existing studies on the reuse of wood waste highlights both substantial potential advantages for sustainability and notable limitations for unsustainable practises. The review revealed diverse end uses that leverage the inherent properties of wood waste, spanning renewable energy, green building materials, adsorbents, and soil amendments. These applications highlight the significant opportunities for wood waste as a future resource. However, the review also revealed that certain mainstream wood waste practices perpetuate issues that are counter to sustainability principles. Specifically, contamination from toxic preservatives, uncontrolled emissions from combustion, unintended impacts on soils, and deficient lifecycle accounting undermine the goals of responsible wood waste management. The foremost priority across all reuse pathways is preventing environmental contamination and harmful exposures by rigorously screening waste wood sources and processing them to remove or destroy hazardous residues. Developing efficient sorting systems and decontamination technologies tailored to wood waste is instrumental in mitigating risks. Strong quality control programs and standards can build confidence in products derived from recycled wood. Life cycle assessment and holistic monitoring of sustainability indicators beyond greenhouse gases are imperative to guide responsible practices. While wood waste shows versatility as a renewable resource, substantial technological innovation, quality control procedures, and supportive policies are still needed to improve economic viability and responsible management on an industrial scale. Continued research addressing technical hurdles, commercial optimization, and environmental monitoring will help wood waste realise its full potential as a sustainable material flow. However, integration across the entire supply chain alongside open communication and cooperation between generators, processors, and end users is equally vital to ensure responsible utilization. With collective commitment from public and private stakeholders, wood waste can contribute to circular economies as populations and industries continue to generate abundant lignocellulosic residuals requiring sustainable futures.

## Supporting information

S1 Checklist (DOCX)

S1 Table (XLSX)

S1 Data (XLSX)
